# Caring for Persons With Intellectual Disabilities and Challenging Behavior: Staff Experiences With a Web-Based Training Program

**DOI:** 10.3389/fpsyt.2021.580923

**Published:** 2021-01-28

**Authors:** Anh Truong, Catrin Alverbratt, Anette Ekström-Bergström, Helena Antonsson

**Affiliations:** ^1^Department of Health Sciences, University West, Trollhättan, Sweden; ^2^Department of Nursing and Reproductive, Perinatal and Sexual Health, School of Health Sciences, University of Skövde, Skövde, Sweden; ^3^Department of Nursing, Umeå University, Umeå, Sweden

**Keywords:** professionals, health and social care services, web-based training, intellectual disability, communication, workplace learning, quality of care

## Abstract

**Background:** Clear and effective communication is a prerequisite to provide help and support in healthcare situations, especially in health, and social care services for persons with intellectual disabilities, as these clients commonly experience communication difficulties. Knowledge about how to communicate effectively is integral to ensuring the quality of care. Currently, however, there is a lack of such knowledge among staff working in the disabilities sector, which is exacerbated by challenges in the competence provision in municipal health and social care services. Therefore, the aim of the study was to explore staffs' experience of web-based training in relation to their professional caring for persons with intellectual disabilities and challenging behavior. The intention is to move toward well-evaluated and proven web-based training in order to contribute to competence provision in this specific context.

**Methods:** Fourteen semi-structured interviews were carried out with individual staff members to gather data regarding their experiences with web-based training in relation to their profession. The collected data were analyzed using qualitative content analysis with a focus on both manifest and latent content.

**Results:** The staff's experiences with the web-based training program were presented as a single main theme: “Web-based training for staff initiates a workplace learning process by promoting reflections on and awareness of how to better care for persons with intellectual disabilities and challenging behavior.” This theme contained three categories which are based on eight sub-categories.

**Conclusion and clinical implications:** The benefits of web-based training for workplace learning could clearly be observed in the strengthening of professional care for persons with intellectual disabilities and challenging behavior. Staff members claimed to have gained novel insights about how to better care for clients as well as about the importance of interactions in their encounters with clients. Professional teamwork is crucial to providing effective care for persons with intellectual disabilities and challenging behavior. Hence, future research aimed at investigating the views of other healthcare professionals, such as registered nurses, is recommended to improve the competence provision within municipal health and social care services and thereby enhance the quality of care.

## Introduction

Persons with an intellectual disability (ID), particularly those who have moderate or severe intellectual impairment, need professional care and support in their everyday lives. This assertion is in accordance with the Swedish Act Concerning Support and Service for Persons with Certain Functional Impairments (1). Functioning communication is a prerequisite to provide help and support in healthcare situations and is a valuable contributor to better outcomes and improved quality of care (2). Poor communication, on the other hand, is perceived to be a barrier to effective healthcare for both healthcare professionals and persons with an ID (3). Therefore, for healthcare professionals, knowledge about how to communicate effectively is integral to ensuring optimal health outcomes (4). However, persons with an ID commonly experience difficulties with communication to varying degrees (5–8). Such communication difficulties are considered to be among the underlying causes of challenging behavior (CB) in persons with IDs (9–11). It has been shown that the severity of CB is usually proportional to the extent of communication difficulties experienced by persons with an ID (7, 9, 10). CB is often an expression of an unfulfilled desire or a manifestation of an unmet need, both of which typically culminate in frustration. Frustration occurring as the result of clients feeling ignored or misunderstood by healthcare professionals is the most commonly reported reason for engaging in CB (12).

CB describes a wide range of behaviors that may be harmful to both the individuals who engage in such behaviors and to those in proximity to these individuals, including healthcare professionals. Examples of CB include aggressiveness, self-injury, non-compliance in care situations, persistent screaming, and overactivity (13). Healthcare professionals who work with persons with IDs report as CB problems behaviors that they frequently witness or experience and/or struggle to manage (14, 15). The inability to address CB in the preferred manner may cause stress, fear, anger, and a sense of powerlessness among healthcare professionals in residential settings and may increase the risk of burnout (15–17). As a consequence, healthcare professionals tend to develop ineffective coping strategies, like avoidance, which in turn can adversely affect their interactions with persons with IDs and thereby degrade the quality of care the professionals can provide (18). Communication and interaction have been mentioned as key factors for ensuring the quality of care providing by healthcare professionals who work with persons with IDs in a residential facility (19). In a review, educational training for healthcare professionals in general communication with persons with IDs was identified as a priority (20). Nonetheless, healthcare professionals do not always have access to such training nor to the resources needed to practice skillful communication when supporting persons with IDs (21).

Persons with IDs value the time they spend interacting with healthcare professionals (22). As such, productive conversations with and attentiveness to persons with IDs could help mitigate or reduce the incidence of difficult situations and emotions (23). Therefore, developing knowledge about effective communication and interaction among healthcare professionals would likely have direct, beneficial impacts on the quality of care for persons with IDs.

In a situation report, both the Health and Social Care Inspectorate (IVO) and the Swedish National Board of Health and Welfare stated that there is currently a lack of knowledge required to provide care for the clients among healthcare professionals working in the sector of disabilities. In addition, challenges concerning the competence provision likely further contribute to the lack of knowledge among healthcare professionals. These knowledge deficiencies have negative consequences for clients (24). As good-quality care has been characterized as being person-centered, equal, and safe, promoting more active participation by clients should be prioritized (4, 25). This contention corresponds with the findings of previous research, which have demonstrated that the capacity to communicate one's own will and fully experience participation can improve the quality of life for persons with IDs who live in residential facilities (19, 26). Yet again, knowledge about how to communicate effectively forms the basis of encounters with clients and the quality of care they consequently receive. Communication deficiencies should therefore be clearly understood and addressed, as otherwise they can jeopardize patient safety (25).

More specifically, training in effective communication and interaction with persons with IDs is particularly valuable for healthcare professionals working in residential settings (21, 27), where persons with IDs typically live. However, in Sweden, municipal health and social care services are currently experiencing a shortage of healthcare professionals in general (28) and healthcare professionals working within residential facilities for persons with IDs in particular (hereafter in this work, those healthcare professionals who work in these facilities will be referred to as *staff*). Unfortunately, as a consequence of such under-staffing and associated economic circumstances (24), enrolling current staff in training at off-site locations would adversely impact the quality of care for clients and would also unduly increase the already substantial workload of non-enrolled staff. Therefore, when planning training programs, a tradeoff between staff training and workload must be adequately considered and addressed. Earlier research has shown that web-based training for staff within healthcare settings can generate good results (29–31). In addition to being able to participate during work hours (31), staff can also overcome access issues in the delivery of training (30).

Internationally, various training programs, for both groups and individuals, have yielded positive outcomes and have had beneficial impacts on the quality of communication between healthcare professionals and persons with IDs (32). In Sweden, a pilot study was conducted on a web-based training program designed to improve the communication and interaction abilities of staff at a residential facility for adults with IDs (33). In this program, a causal modeling approach was applied as the design and evaluation framework (34). The content of the web-based training program was based on a theoretical model that included the assumption that CB is caused by interrupted communication between staff and persons with IDs. By raising awareness and enhancing understanding among staff members of their own values, emotional reactions, skill repertoires, and thoughts concerning individuals with IDs, as well as by expanding their perception of the physical environment and workplace culture in which they operate, the likelihood of achieving and sustaining effective and productive interactions with clients was expected to increase (35). In the pilot study it was shown that web-based training produces beneficial outcomes and is greatly appreciated by the participants (33). Given these previous results, conducting a larger study based on the knowledge derived from the results would greatly benefit communication and interaction between staff and clients with IDs and CB. It is for this reason that the present study was developed and carried out. More precisely, this study sought to facilitate a deeper understanding of staff experiences with web-based training in relation to caring for persons with IDs and CB. In addition, it was hoped that this study would contribute to the further development of the web-based training program with the ultimate aim of achieving more individualized and better-quality care for persons with IDs and CB in the health and social care settings. Therefore, the aim of the current study was to explore staff experiences with a web-based training program in relation to their professional care for persons with intellectual disabilities and challenging behavior.

## Methods

### Design

In order to accomplish the study's objective, a qualitative and inductive approach was applied. Data collection was carried out via individual semi-structured interviews. The collected data were assessed by conducting qualitative content analysis with a focus on both manifest and latent content (36).

### The Web-Based Educational Intervention

The web-based training program consisted of a text section and a streamed-lecture section. The construction of the program proceeded from the same basis used to develop the pilot study (33). In the pilot study, the web-based training program was assessed in terms of several topics, e.g., definitions and characteristics of IDs and CB, as well as their possible causes and consequences, including deteriorated mental health. Likewise, the importance of communication and interaction, in addition to the availability of coping and prevention efforts, were highlighted and discussed with regard to the quality of care and support. Similarly, focus was placed on the values, feelings, and abilities of the staff themselves, especially with respect to the workplace culture and physical environment in relation to the development of CB in persons with IDs. To stimulate reflections, cases of encounters between staff and persons with ID and CB were included (33).

The present study differed from the pilot study described above in its enhanced emphasis on streamed lectures and in-depth scientific scrutiny aimed at enhancing learning for a multi-professional group. Both the text section and streamed-lecture section covered a range of knowledge, from basic information to more theoretical discussions and reasoning linked to relevant scientific findings within the area. In total, 12 streamed lectures were included, varying in duration from 4.5 min to 14 min. The text section consisted of three chapters, each of which ended with opportunities for self-reflection. The participants were encouraged to share and discuss these self-reflections with co-workers. Lastly, they were given access to previous research in the area.

### Setting

A total of 20 residential facilities for persons with IDs, aged 18 and over, in a medium-size city in Sweden were included in the current study. The staff employed in these facilities worked weekdays as well as weekends, including daytime, evening, and nighttime shifts. The individuals who lived in the facilities (referred to hereafter as *clients*) had access to staff around the clock for support and assistance in structuring their everyday lives, e.g., when to work, when to pursue other daily activities, and when to do housework. Depending on how many clients lived in the residential facility, the severity of their ID, and the time of day, there were between one to six staff members on duty at any time. Most of the facilities had an office with one or two computers, which were used mainly for documentation. These computers were also intended for use in the web-based training program. Some facilities did not have an office; in these facilities, the staff carried out the web-based training in a breakroom.

Most of the facilities held regular staff meetings, e.g., every 2 weeks, with the service manager to discuss ongoing work activities. Some facilities offered a web-based introduction for new employees; otherwise, no regular training programs for the staff were conducted.

### Procedure and Data Collection

Two of the co-authors (CA and HA) attended the workplace meetings in which the web-based training program and subsequent interviews were introduced. All staff members who were permanently employed in the facilities were offered the web-based training program during their work hours. Some facilities even offered this training to temporary employees. The web-based training occurred from spring 2018 until autumn 2018 after receiving approval from the administration manager and from the service managers of the residential facilities.

After completing web-based training, the participants were once again asked, verbally during staff meetings and in writing by email, to participate in an interview. The informants were either working in the residential facilities or were responsible for providing education and supervision concerning workplace issues with respect to persons with IDs and CB. Fourteen informants (11 women and 3 men) aged 27–55 agreed to participate in the study. Of the nine informants who had received upper secondary school education, five had specialized in the care of persons with IDs. More precisely, one informant had received higher vocational education, whereas the remaining four had received university education in the social sciences. The work experience with persons with IDs ranged from 8 to 30 years.

The semi-structured interviews were conducted by the first author (AT) and two of the co-authors (CA and HA) at a location chosen by the informants. The interviews were based on two open-ended questions: “*What is your experience of attending the web-based training?”* and “*What do you think about the web-based training in relation to your daily work with persons with IDs and CB?”* Follow-up questions were directed in such a way as to encourage the staff to freely share their experiences. All of the interviews were recorded with the permission of the informants. The duration of the interviews ranged from 30 to 77 min (mean = 53 min).

A pilot interview was used to test the efficacy of the interview guide. The results of the pilot interview indicated that only minor changes needed to be made to the interview questions to facilitate more in-depth depictions. Accordingly, the pilot interview and its findings were included in the final analysis.

### Analysis

The interviews were transcribed verbatim by the first author (AT). The transcriptions were read as the corresponding audio files were simultaneously played in order to cultivate a better sense of what the informants were seeking to express. Thereafter, the transcriptions were reviewed several times in order to ensure that the full content of the text was comprehensively assessed and its findings were fully elicited (36).

In the following step, all statements that corresponded to the aim of the study were underlined. Meaning units were identified before being condensed and were subsequently labeled with codes, which in this case constituted brief descriptions or essential summaries of the statements. The created codes were first discussed among all the authors and then further developed in relation to the original text, if needed. In the next step, the codes were collated with regard to similarities and differences and were accordingly grouped into subcategories. All of the codes within a subcategory were connected to a common denominator, which was assigned as the name of that subcategory. From these subcategories, the categories were created that represented the manifest content of the data. The analysis process consisted of several iterative steps by which an understanding of the data was gained. Throughout the analysis process, attempts were made to capture the latent content of the data, which was represented by a theme corresponding to the aim of the study (see Table 1). The generation of the categories, and single main theme was accomplished cooperatively by all authors.

**Table 1:** Categories with subcategories and theme as they emerged from interviews with the staff.

**Subcategory**	**Category**	**Theme**
Independence Individual responsibility Fundamental prerequisites	Web-based training provides freedom but also requires responsibility, both of which affect the learning outcome	
Stimulation of reflections and discussions within the work group Structured planning to enhance learning	The learning process contributes to generating insights about caring through reflection	Web-based training for staff initiates a workplace learning process by promoting reflections on and awareness of how to better care for persons with IDs and CB
Gaining of awareness and understanding about caring for persons with IDs and CB The pedagogical arrangement Perceptions and attitudes about training	The mutual impact of training and the opinions of staff about learning for the care of persons with IDs and CB	

### Ethical Considerations

Approval to conduct the study was obtained from the Swedish Ethical Review Authority, Dnr 35517. In the study, ethical principles were followed in accordance with the Helsinki Declarations (37). Ultimately, the study provided staff with the opportunity to acquire valuable knowledge that would support their work with persons with IDs and CB, thereby improving the clients' quality of care. The facilities and their staff were provided with all necessary information about the research in writing as well as verbally. Participation was voluntary, and written consent was required from participants before their involvement in the study. The confidentiality of each informant was ensured by removing any information from the data that could be used to identify the participants.

## Results

The staff's experiences with the web-based training program in relation to their professional care for persons with IDs and CB were presented as a single main theme: “*Web-based training for staff initiates a workplace learning process by promoting reflections on and awareness of how to better care for persons with IDs and CB.”* This theme contained three categories: “*Web-based training provides freedom but also requires responsibility, both of which affect the learning outcome,” “The learning process contributes to generating of insights about caring through reflection,”* and “*The mutual impact of training and the opinions of staff about learning for the care of persons with IDs and CB.”* These categories were based on eight sub-categories (Table 1). Each category and associated subcategories are presented in this work by using quotations to illustrate the subjective experiences of the staff.

### Web-Based Training Provides Freedom but Also Requires Responsibility, Both of Which Affect the Learning Outcome

This category encompassed informants' thoughts about taking part in web-based training during work hours. The informants appreciated the freedom they had to organize their own training sessions. However, they also realized the magnitude of demands placed upon them, as well as their responsibility to complete the web-based training, provided they had access to a device and were reasonably digitally literate.

#### Independence

The informants appreciated having the freedom to organize their own training sessions; for example, they did not have to be in any particular place to take part in them; a web-enabled digital device was all they needed to obtain access to the training. They also felt free to complete the training at their own pace. As the informants did not need to take other learners into consideration and were not faced with disturbances by others, they could regulate their own training under completely different conditions as compared to a normal classroom education. As one informant mentioned:

“And if it is a (classroom) lecture that just goes on like this, then you can't pause or go back to write things down, then. you can miss a lot.”

Under traditional training circumstances, informants might sometimes not feel like they wanted to attend courses due to heavy or otherwise incomplete workloads, which would have left them frustrated by the lack of time needed to take part in the course. Being able to choose when to complete the training thus increased their motivation to do so, thereby providing them with better opportunities to implement their training. As one informant remarked:

“Ok, now I cannot take this part, so now I can focus on these here. And then you will go into it wholeheartedly.”

This independent nature of the training also enhanced access to the program for the whole work group, meaning that everyone had the same likelihood of acquiring the requisite knowledge. Accordingly, independence was considered to be beneficial from these points of view.

#### Individual Responsibility

Individual responsibility was required to complete the training. In other words, each informant had to develop their own plan for completing the training and for ensuring that they finished the training based on their own initiative. One informant described this unspoken responsibility as follows:

“No one telling you what to do. when to do it. You got to take it when you know you actually can…”

The informants drew up a plan in agreement with their co-workers and took turns completing the training program. They each had to find moments in which they could participate in the training, which often took time. It was about undertaking and finishing the training according to each person's own responsibility.

#### Fundamental Prerequisites

Some technical concerns were raised about the training. Although most of the informants were familiar with web-based training, some had never before engaged in such training and consequently did not feel very comfortable with computers, requiring additional effort and time to address. During the training, these informants had to ask for help from their younger co-workers. One informant described this feeling as being a burden to others:

“There were some. young people who were high-tech. They just, chop chop! It was almost done, you know? Pressing here and there. and another almost got sweating of the hands, you feel. sweat on your forehead, you know? And you need to ask for help…”

Additionally, some informants also mentioned that it was difficult to find a peaceful place to train and/or gain access to a digital device at the workplace, as most facilities only had one computer for the entire work group to share.

### The Learning Process Contributes to Generating Insights About Caring Through Reflection

With the training, the informants were given opportunities for self-reflection and discussion of these self-reflections with other co-workers. They also had the chance to express their thoughts about daily work concerning the care of clients and to receive fresh insights as a result. At the same time, requests were made for additional group discussions, and the desire for better planning to enhance learning among the staff was expressed.

#### Stimulation of Reflections and Discussions Within the Work Group

Being able to undertake training during work hours was considered beneficial. The participants could pause to consider and express their thoughts about their daily work, often resulting in the generation of novel insights concerning various work aspects. Training amidst everyday work facilitated self-reflection and made it easier to connect reflections and instructions from the training to their actual job tasks. This connection was emphasized by one informant:

“When you do a web training, you usually do it in daily work. Then, reflection may occur more easily.”

Furthermore, the training was conducive to encouraging reflections and spontaneous discussions about how to best care for clients even within the work group. The informants discussed various training lessons and insights with each other, which in turn prompted interesting and fruitful conversations. By having such discussions with co-workers, the informants could each learn from each other, thereby reinforcing their cohesion and solidarity as a work group.

#### Structured Planning to Enhance Learning

Comments were made about the need for more supportive planning from the organization to enable training and learning. Further, emphasis was placed on the need for a clearer and more effective training framework. This is because not all of the informants had sufficient time to complete the training. Even when time was allocated by organizations, it differed in duration among the units. Therefore, it was suggested that the training be better planned and streamlined with respect to work schedules; doing so would, according to the informants, help them to stay focused when participating in the training. Moreover, the planning of follow-up training as well as its actual implementation should be more overt, providing equal time for collaborative work and individual training, which would help increase commitment to learning. The absence of follow-up training was noted by one informant:

“*The training has a prerequisite that is not used by the workplaces. I have talked to others; no one has felt that you are discussing it in different groups, maybe in a workplace meeting. I think it's sad, I think it is wasteful if you don't…”*

The informants emphasized the importance of sharing their experiences with others in a collaborative setting. They believed that knowledge can spontaneously emerge in such settings, and that collaborative discussions might lead to unique combinations of knowledge.

### The Mutual Impact of Training and the Opinions of Staff About Learning for the Care of Persons With IDs and CB

This category included informants' reflections on the impact of the training on themselves as well as their opinions about the learning required for completing their daily work with respect to caring for clients. The informants were pleased that issues pertaining to difficulties they experienced in their daily work with clients had been brought to light. Their increased understanding of clients also made them feel more confident in their encounters with the clients. The arrangement of the training components and the relative amount of time allocated to each were considered well-balanced and appropriate. That said, some were of the opinion that the training could be extended to cover a greater range of needs on various levels. Likewise, there was agreement that the content and lessons provided by the training could be more tangible.

#### Gaining of Awareness and Understanding About Caring for Persons With IDs and CB

The training and its set-up encompassed the knowledge needed by the informants to handle the types of CB they faced in their everyday work. The informants felt strengthened by the training, as it gave them the opportunity to review their previous knowledge. The level of the training program was considered acceptable for new temporary staff, and even for staff originating from foreign countries, for whom it was regarded as especially valuable. The training could then be more effectively absorbed. All of the informants received the exact same content and knowledge, which they used to discuss and argue about their different views, about which one informant remarked:

“At my level, I think it's just right; it's just good for me. We are different. I have Swedish as the second language.”

Since the training, problems in their daily work have inevitably surfaced, and the informants have expressed appreciation that their profession was finally receiving adequate attention. Their feeling of being exposed to violence in their daily work, for example, was validated. They gathered the courage to talk openly about it, such as the following informant:

“… and I think that is part of the training, that it is. that you dare to lift it. I feel that way.”

After undertaking training, the informants' understanding of client behaviors was enhanced. They realized that some of these behaviors were not caused by them, by what they had said or done in problematic situations. In this way, they felt more confident about their encounters with clients. Their increased awareness and understanding also changed the ways in which they worked with the clients.

The staff desired training so they could acquire greater knowledge and receive advice that would help them identify concrete solutions to existing problems in the daily care, for instance problem with self-harm. They also wanted to learn additional strategies that would help their clients. This concern was stressed by one informant:

“… as he who banged his head against the wall and the wardrobe doors,. if you could find some strategy to change that behavior to something else… less harmful…”

The informants were hopeful for more training as a result of experiencing web-based training first-hand.

#### The Pedagogical Arrangement

The training arrangement and its various components were considered to be well-balanced and appropriate. Likewise, the design offered many ways to assimilate the training which was considered pedagogically. Some informants found that pleasant simply sitting there and listening to the streamed-lectures while others preferred to read the texts by themselves. At the same time, concerns were raised about the content and lessons, which informants thought should be more tangible. A concern was also raised about the presentation of the content, which some informants thought should be better designed to capture the interest of participants and provoke novel thoughts and curiosity. The informants preferred for the lecturer to speak more vividly about the topic and not place too much text on a PowerPoint presentation. An alternative, such as showing some pictures to better explicate the lessons, was considered.

Some informants expressed the desire for a better division of training components and a reduction in the number of components overall. Doing so would, according to these informants, make it easier to accurately perceive various aspects of the training. Take the following thought expressed by one informant:

“… and you have finished this part and still have all these left… you almost get tired of seeing how much you have left to go through… the movies talk about the same thing a lot; why not merge into a few instead of so many?”

Moreover, mixed activities, e.g., text and streamed lectures, could be better integrated with some reflection questions or cases. Likewise, including more concrete issues associated with the daily work with which informants engage would help them to understand underlying theory and connect it with their job duties.

#### Perceptions and Attitudes About Training

Some informants expressed concern that the training did not meet their needs and expectations. These informants went on to state that they were not concerned about potential threats and violence in their residential facility and therefore did not require training on such issues. As one informant commented:

“I think the arrangement was great. You could certainly have used it, if we had a lot of threats and violence, then you can work with that,… but right now we do not have that… So it is a little difficult. Otherwise, it was great.”

Volition and interest on the part of individuals were essential, as was the opportunity to be involved. Some informants claimed that they already possessed sufficient information about the topics presented in the training, as they had been working in the sector for years. Sitting in front of a computer was tiring, some said, but they simply had to accept the development. Some informants went on to reveal that the staff tended to get stuck in their work methods, and that not everyone was comfortable with the changes.

### Web-Based Training for Staff Initiates a Workplace Learning Process by Promoting Reflections on and Awareness of How to Better Care for Persons With IDs and CB

Overall, the web-based training program for staff initiated a workplace learning process by promoting reflections on and awareness of how to better care for persons with IDs and CB. The staff claimed that they had gained novel insights into the profession and into the caring process for persons with IDs and CB. Opinions about clients and CB changed somewhat, and the staff were inspired to adopt new ways of working. For instance, they allowed themselves to take a step back, giving clients the room and time to express themselves when they were experiencing an emotional outburst. At the same time, the explicit need for prerequisites, such as access to a device and learning-supported planning, was expressed. Furthermore, staff members called for the expansion of training to better meet the needs of staff, such as methods for dealing with clients who engage in self-harm. Also, informants claimed that more effort was needed to work with the understandings and the attitudes of staff, and that the importance of learning and knowledge as integral components in the care of clients should be further emphasized. Healthcare organizations could play a key role in this process by announcing and encouraging training, which could in turn promote greater commitment to training among their staff. These factors were considered essential to training and to assisting staff in acquiring a more useful knowledge base.

## Discussion

The aim of the present study was to explore the experiences of staff with a web-based training program in relation to their professional care for persons with IDs and CB. Our findings showed that the program initiated a workplace learning process by promoting self-reflections and awareness of how to better care for persons with IDs and CB. The staff claimed that they gained novel insights concerning the care of their clients. For instance, some staff members realized that stepping back and giving clients room and time to express themselves when they are experiencing an emotional outburst can ultimately benefit interactions. They were also inspired by the new knowledge they obtained concerning the dynamics underlying CB. The web-based training program strengthened the ability of staff to meet the individual needs of persons with IDs and CB. Furthermore, staff members called for the expansion of training to better meet the needs of staff, such as methods for dealing with clients who engage in self-harm. Also, informants claimed that more effort was needed to work with the understandings and the attitudes of staff, make it clear that learning and knowledge were integral to the care of clients and should as such be further emphasized. Healthcare organizations could play a key role in this process by announcing and encouraging training, which could in turn promote greater commitment to training among their staff.

The results of this study demonstrated how staff members realized that stepping back and giving clients room and time to express themselves when they are experiencing an emotional outburst can ultimately benefit interactions. Communication and interaction have been mentioned as key factors for healthcare professionals who work with persons with IDs in a residential facility, ensuring the quality of care (19). Therefore, educational training for staff in general communication with persons with IDs is a priority (20). In addition, one prior study revealed that CB could predict emotional exhaustion and was correlated with increased stress and burnout rates among staff, which were in turn associated with relative levels of commitment by staff to their work. Therefore, the importance of interventions that enable staff to better manage CB among their clients should be stressed (14). This emphasis is congruent with findings from another study on nurses who work with people with IDs (38).

The informants reflected on and reasoned about the opportunities and challenges entailed by their work and with regard to web-based training. Additionally, they mentioned benefiting from many novel insights about the mutual impact between training and staff members' own opinions in relation to attaining knowledge required for their profession. These aspects are further discussed below.

In this study, web-based training seemed to have had a stimulating effect on workplace learning. Carrying out the training in the workplace triggered self-reflections on and discussions within the work group that generated insights into their own profession. The results of this study also showed that more effort was needed to understand the attitudes of staff, and that it was important to stress the integrality of learning and knowledge in the care of clients. Healthcare organizations could play a key role in this process by announcing and encouraging training, which could in turn promote greater commitment to training among their staff. Van Woerkom and Poell stated that workplaces constitute a learning environment due to the opportunities they offer for combining formal education with informal learning, individual efforts, and teamwork. Furthermore, workplaces enable exchanges between novices and experts (39). By participating in activities and interactions in the workplace, various kinds of ongoing learning arise (40). Learning comprises two elements: a social process that occurs in interaction with the environment, and an inner psychological process that transpires within individuals (41). Hence, sharing self-reflections with group members in addition to individual study is essential for stimulating and consequently extracting knowledge from training. This process was also demonstrated in another study in which the outcome of web-based training in combination with group-based discussions was compared to web-based training alone. Both groups gained significant knowledge from the training. The group that, in addition, could discuss the client scenarios of its members and continue to promote its most productive ideas reported having better training outcomes and improved practices compared with the group that experienced only web-based training (42). This demonstrates the importance of both individual processes and larger interactions with one's social surroundings, a consideration that should be included in planning as well. In this study, close attention was paid to how to enable both individual and the social processes but, judging from the results, further developments should focus on optimizing the effect of social interaction. Arranging discussions with supervisors to clarify the connections between the training and the daily practices could increase learning among staff even further.

As our results showed, the independence offered by web-based training made it easier for many informants to complete their training. A similar result was also found in an earlier study that revealed the flexibility of web-based courses in allowing participants, especially adult learners, to choose when to start and complete certain assignments (43). At the same time, web-based training places greater emphasis on the personal responsibility and accountability of participants. This dimension was illustrated by Federman (44), who stated that e-learners are free to decide the pace and timing of their training and are as such ultimately key to their own training. As the web-based training in the current study was completely self-regulated, it was crucial for participants to be willing and motivated to take responsibility for themselves in their training. For some, it was not easy to use a computer; hence, their training did not proceed as smoothly as it did for other informants. Furthermore, access to the devices needed for the training was not always optimal in the workplace. In this respect, there have been warnings about the implications of the unrestricted introduction of information technology into the educational context in recent years. In this view, greater use of information technology in educational settings will not necessarily improve the quality of education—quite the opposite, technology is likely to exacerbate social inequality by disenfranchising those with less access to digital devices and/or less training in the use of such technology (45). Our results indicate that far more attention should be paid to overcoming the abovementioned obstacles. Doing so would especially benefit staff within municipal residential care services. Besides, as shown by our results, not everyone prefers web-based learning. Other studies have indicated that students who like web-based learning will likely receive more effective instruction than those who dislike it. Thus, each student's preferred mode of learning should be taken into consideration (46, 47). We should keep this in mind when offering competence provision by way of web-based training. Nevertheless, the informants in the present study agreed that the job of caring for persons with IDs is not easy to perform, and that more knowledge is thus needed in health and social care services. The informants also found it regrettable that accessibility to education and training within the sector has been reduced in recent years due to financial circumstances. Finally, research on designing learning through supportive web-based lectures has only just begun (48), and as such it would be wasteful not to look for opportunities to make use of the potential of such technology in competence development for the benefit of health and social care services in municipalities.

The informants in this study shared their thoughts about web-based training in relation to their own needs and everyday work. Some found the training to be helpful, strengthening them as professionals and making them more confident in their encounters with clients. In another study, reflections, and discussions in connection with training were considered to be related to local resources, beliefs, and methods participants would likely choose to improve work pathways (42). These considerations are in agreement with the basic assumptions concerning web-based training in the current study. One assumption was that by working with the staff's own values, and when accompanied by the cultural and skill repertoires of the staff, interactions with clients would be improved (35). This effect was identifiable in statements made by the informants in our study. The informants appeared to be more confident in their encounters with clients, and their interactions with clients with regard to the basic conditions of person-centered care and the fundamental idea of promoting clients' participation in and influence over their own care were enhanced (25). Person-centered care is based on the fact that clients are people who have their own wills, abilities, and needs. In addition, the capacity to personally relate with clients, i.e., forming a partnership with clients for the furtherance of care, has been emphasized (49). Fredriksson proposed that communication in a care context is the basic prerequisite for understanding the patient's experience of their own reality. Hence, it has enormous significance in a complex world. Besides, communication enables the development of productive relationships between staff and their clients, which in turn permits a higher degree of well-being for both parties (50). Persons with IDs value the time they spend interacting with healthcare professionals (22). As such, productive conversations with and attentiveness to persons with IDs could help mitigate or reduce the incidence of difficult situations and emotions (23). Confident encounters would constitute the foundation of such partnerships, in which clients could receive the support they need and staff could provide the support as intended, which would in turn cultivate the partnership even further. Consequently, it is our opinion that web-based training can create the partnerships required for person-centered care. The provision of person-centered care may also culminate in better well-being and job satisfaction among professionals working with persons with IDs (51). These researchers remind us that considering the welfare of healthcare professionals is equally crucial, as they might otherwise experience significant work stress and increased rates of burnout (51).

In contrast, concerns were raised in this study about the quality of training, which did not meet some informants' expectations. Accordingly, suggestions were made for improving the design and procedures of the training. Some informants believed that they did not need the topical knowledge offered by the program, or they were already well-informed about such topics. However, Damschroder et al. argue that the capacity to meet the different knowledge needs in large, heterogeneous groups which itself is a challenge. The complexity of this issue shows to increase as the number of potential organizational targets or people increase (52). This finding is in line with the results and contexts of the present study. Even though the units were all municipal residential facilities for adults with IDs, they nonetheless diverged with respect to direction of ID. The clients also varied in terms of age, severity of ID, and so on, meaning that the extent and severity of CB also differed widely among them. Furthermore, differences with regard to staff members' backgrounds and predispositions, e.g., education, work experience, sense of responsibility and motivation, can also play a key role in this connection. Fulfilling all the requests made within a single training session might be difficult, and as such further development and customization of the training program is warranted.

Nonetheless, knowledge is, to a certain extent, about cognitive growth, which depends not just on knowing more but also on restructuring what is already known in order to make connections with new forms of knowledge (53). Moreover, knowledge does not exist in objects or events themselves but rather only emerges in our descriptions or analyses of such objects or events (54). Based on these arguments, knowledge generated from the web-based training program in this study constituted knowledge only to the extent that participants actively, cognitively worked with it and established connections between it and (restructured) prior knowledge, which is known as the acquisition process (41). Only then does progress in learning truly arise. Without these consciously applied activities, there would only be “*a lot of trees but no wood”* (53). Further, learning demands the mobilization of mental energy. Everything we learn also has an emotional side, on which both the acquisition process and the learning outcome depend (41). Illeris propose in his research that adults typically react with resistance to learning when they are faced with pressure or forced into learning courses for which they subjectively cannot see the point or in which they have no interest, or when other, subjectively unacceptable conditions apply (41). This statement is in accordance with the findings by Luceys study that shows that useful knowledge applicable to the learner's current career, along with interesting course materials relevant to real-world issues, are motivating factors for adult learners to actively pursue training (43). In addition, organizational supports appear to be relevant for improving learning outcomes (42). Previous studies have illustrated significant differences in perceived workplace learning support via participant reports from different occupational groups. Higher-status occupations offer a workplace environment that is more conducive to learning than that of lower-status occupations (55). These aspects must be addressed and overcome to fully develop the competence provision and counteract potential negative consequences. Only then can the quality of care in municipal health and social care services for persons with IDs and CB be substantively improved.

### Methodological Considerations

Efforts were made to ensure the trustworthiness of the research data by abiding by the three crucial aspects of *credibility, dependability*, and *confirmability* in the different stages of the study. Interviewing individuals who have first-hand experience with a phenomenon and are thus able to accurately discuss it in detail increases the *credibility* of the study (56). Likewise, choosing informants of various genders, ages, and experiences can more comprehensively address the research questions by approaching them from a variety of angles (36). In this study, all of the informants, who constituted a mix of men and women, participated in the web-based training program. One informant was also of foreign descent. The informants were of mixed age, represented a variety of educational levels, and had varied durations of work experience with persons with IDs. As such, this sample might reflect contemporary variations in the current healthcare sector. Interview questions were first tested through a pilot interview, which has been described as enhancing a study's *credibility* (57). The fact that the interviews were conducted by three researchers could be considered a limitation in terms of *dependability*, as follow-up questions could have been asked in various ways depending on the divergent subjective traits of each researcher. Conversely, employing three researchers may have in fact captured useful variations in the experiences of the participants (58). Similarly, throughout the analysis process, regular discussions on various issues were held between all researchers in the author group until consensus was reached. This process was undertaken to manage our pre-understandings and mitigate our underlying subjective biases, thereby preventing them from adversely impacting our interpretations (56). Such pre-understandings and biases emerge out of lessons and experiences tied to one's profession, subject area, educational background, and other types of training. Therefore, assembling a multidisciplinary team helped us to manage such underlying assumptions. Ultimately, it is the voice of the participants that should be emphasized, as doing so greatly improves the *dependability* of the study. All authors were involved in the analysis of the results in this study. The transcriptions were read as the corresponding audio files were simultaneously played in order to cultivate a better sense of what the informants were seeking to express. We took notes, for instance, about silence, laughter, and gestures, which were included in our interpretations of the informants' statements. Doing so likely enhanced the *confirmability* (57) of our research. While organizing and grouping the data into subcategories and categories, the first author (AT) repeatedly reviewed all the previous steps to identify all meaning units, sometimes even in the original interview text. This was done to ensure, as much as possible, that the content was not taken out of context, which is a consideration to which a researcher should pay particular attention during the analysis process (58).

An interview is an exchange of views between two persons who talk about a topic of mutual interest; the knowledge that consequently arises is thus constructed in and by the interaction between these parties (58, 59). The topic in this case was the evaluation of web-based training, about which the informants shared their experiences in interviews with the researcher who developed the training program. This knowledge could have been perceived as a mental obstacle to the informants, who may not have felt free to express themselves fully. To overcome this obstacle, strenuous efforts were made to encourage the informants to share their experiences with training, both positive and negative, as honestly and comprehensively as possible.

## Conclusions and Clinical Implications

The benefits of web-based training for workplace learning clearly included the strengthening of professional care for persons with IDs and CB. The staff claimed to have gained novel insights about how to care for clients as well as about the importance of interaction in encounters with their clients. For instance, they allowed themselves to take a step back, giving clients the room and time to express themselves when they were experiencing an emotional outburst.

Our findings illustrate the complexity of providing staff training for the workplace through web-based training program. Beyond the benefits of web-based training for workplace learning, some challenges also emerged. We conclude that web-based training, workplace organization, and individuals' opinions each have an important impact on the learning outcome. To reach the best possible outcome, however, resources need to be invested in all three parts concurrently. This knowledge can contribute to the development of competence provision in municipal health and social care services more generally, where similar circumstances in terms of a notable downward trend in competence provision prevail, a pattern which could ultimately jeopardize the quality of care.

In addition to knowledge, cooperation in both healthcare and social services was also highlighted in order to improve care for persons with IDs and CB, by Swedish Association of Local Authorities and Regions (60). In order to better meet their needs, professional teamwork is critically important. Hence, future research should investigate the views of other healthcare professionals, e.g., registered nurses, to further improve the competence provision within municipal health and social care services and thereby improve the overall quality of care. This approach would enrich our knowledge and understanding of how the competence provision could be enhanced to provide the best possible care for vulnerable groups in our society.

## Data Availability Statement

The datasets presented in this article are not readily available because of the participants' requests for confidentiality. Requests to access the datasets should be directed to correspondent of this article.

## Ethics Statement

The studies involving human participants were reviewed and approved by Swedish Ethical Review Authority, Dnr 35517. The patients/participants provided their written informed consent to participate in this study.

## Author Contributions

AT contributed to data collection, data curation, data analysis and interpretation, as well as to drafting the article, critically revising the article, and obtaining final approval of the version to be published. CA and HA contributed to the conception of the work, to data collection, to data analysis and interpretation, to critical revision of the article, and to obtaining final approval of the version to be published. AE-B contributed to data analysis and interpretation, to critical revision of the article, and to obtaining final approval of the version to be published. All authors contributed to the article and approved the submitted version.

## Conflict of Interest

The authors declare that the research was conducted in the absence of any commercial or financial relationships that could be construed as a potential conflict of interest.

## Abbreviations

ID: Intellectual disability
CB: Challenging behavior.

## References

[1] SFS 1993:387. Act Concerning Support and Service to Persons with Certain Functional Disabilities.

[2] Johnsson A Boman Å Wagman P Pennbrant S. Voices used by nurses when communicating with patients and relatives in a department of medicine for older people—An ethnographic study. J Clin Nurs. (2018) 27:e1640–e50. 10.1111/jocn.14316 29493834

[3] Bell R Does he have sugar in his tea? Communication between people with learning disabilities, their carers and hospital staff. Tizard Learn Disabil Rev. (2012) 17:57–63. 10.1108/13595471211218712

[4] Rubinelli S Silverman J Aelbrecht K Deveugele M Finset A Humphris G. Developing the International Association for Communication in Healthcare (EACH) to address current challenges of health communication. Patient Educ Couns. (2019) 102:1217–21. 10.1016/j.pec.2019.01.004 30661729

[5] Baker V Oldnall L Birkett E McCluskey G Morris J Adults with learning diabilities (ALD): Royal College of Speech and Language Therapists Position Paper. London (2010).

[6] Belva BC Matson JL Sipes M Bamburg JW. An examination of specific communication deficits in adults with profound intellectual disabilities. Res Dev Disabil. (2012) 33:525–9. 10.1016/j.ridd.2011.10.019 22119701

[7] Smith M Manduchi B Burke É Carroll R McCallion P McCarron M. Communication difficulties in adults with intellectual disability: results from a national cross-sectional study. Res Dev Disabil. (2020) 97:103557. 10.1016/j.ridd.2019.103557 31874425

[8] Sutherland D van der Meer L Sigafoos J Mirfin-Veitch B Milner P O'Reilly MF Survey of AAC needs for adults with intellectual disability in New Zealand. J Dev Phys Disabil. (2014) 26:115–22. 10.1007/s10882-013-9347-z

[9] Bowring DL Totsika V Hastings RP Toogood S Griffith GM. Challenging behaviours in adults with an intellectual disability: a total population study and exploration of risk indices. Br J Clin Psychol. (2017) 56:16–32. 10.1111/bjc.12118 27878840

[10] Emerson E Kiernan C Alborz A Reeves D Mason H Swarbrick R. The prevalence of challenging behaviors: a total population study. Res Dev Disabil. (2001) 22:77–93. 10.1016/S0891-4222(00)00061-5 11263632

[11] NICE NIfHaCE. Challenging Behaviour and Learning Disabilities: Prevention and Interventions for People With Learning Disabilities Whose Behaviour Challenges (2015). 26180881

[12] Griffith GM Hutchinson L Hastings RP. “I'm not a patient, I'm a person”: The experiences of individuals with intellectual disabilities and challenging behavior—A thematic synthesis of qualitative studies. Clin Psychol. (2013) 20:469–88. 10.1111/cpsp.1205324105755

[13] Emerson E. Challenging Behaviour: Analysis and Intervention in People with Severe Intellectual Disabilities. Cambridge: Cambridge University Press (2001). 10.1017/CBO9780511543739

[14] Smyth E Healy O Lydon S. An analysis of stress, burnout, and work commitment among disability support staff in the UK. Res Dev Disabil. (2015) 47:297–305. 10.1016/j.ridd.2015.09.023 26469377

[15] Lundström M Åström S Graneheim UH. Caregivers' experiences of exposure to violence in services for people with learning disabilities. J Psychiatr Ment Health Nurs. (2007) 14:338–45. 10.1111/j.1365-2850.2007.01081.x 17517024

[16] Nikku N Bostad Med Särskild Service Och Daglig Verksamhet: En Forskningsöversikt. Stockholm: Socialstyrelsen, (2011). Report No.: Contract No.: 2011-2-6.

[17] Panicker AS Ramesh S. Psychological status and coping styles of caregivers of individuals with intellectual disability and psychiatric illness. J Appl Res Intellect Disabil. (2019) 32:1–14. 10.1111/jar.12496 29947458

[18] Leoni M Alzani L Carvevali D Cavagnola R Chiodelli G Corti S. Stress and wellbeing among professionals working with people with neurodevelopmental disorders. Review and intervention perspectives. Ann Ist Super Sanita. (2020) 56:215–21. 10.4415/ANN_20_02_11 32567571

[19] Kåhlin I Kjellberg A Hagberg J-E Staff experiences of participation in everyday life of older people with intellectual disability who live in group homes. Scand J Disabil Res. (2015) 17:335–52. 10.1080/15017419.2014.941923

[20] Hemm C Dagnan D Meyer TD. Identifying training needs for mainstream healthcare professionals, to prepare them for working with individuals with intellectual disabilities: a systematic review. J Appl Res Intellectual Disabil. (2015) 28:98–110. 10.1111/jar.12117 25266406

[21] Dalton C Sweeney J Communication supports in residential services for people with an intellectual disability. Br J Learn Disabil. (2013) 41:22–30. 10.1111/j.1468-3156.2011.00717.x

[22] Dodevska GA Vassos MV. What qualities are valued in residential direct care workers from the perspective of people with an intellectual disability and managers of accommodation services? J Intellect Disabil Res. (2013) 57:601–15. 10.1111/j.1365-2788.2012.01565.x 22563721

[23] McKenzie K Whelan KJ Mayer C McNall A Noone S Chaplin J “I feel like just a normal person now”: an exploration of the perceptions of people with intellectual disabilities about what is important in the provision of positive behavioural support. Br J Learn Disabil. (2018) 46:241–9. 10.1111/bld.12236

[24] Swedish National Board of Health and Welfare Care and Services for Persons with Impairments, Situation Report 2020. Swedish National Board of Health and Welfare (2020).

[25] Swedish National Board of Health and Welfare Equal Health, Care and Welfare. Swedish National Board of Health and Welfare (2018).

[26] Gur A Challenging behavior, functioning difficulties, and quality of life of adults with intellectual disabilities. Int J Dev Disabil. (2018) 64:45–52. 10.1080/20473869.2016.1221233 PMC8115461 34141290

[27] Bigby C Beadle-Brown J. Improving quality of life outcomes in supported accommodation for people with intellectual disability: What makes a difference? J Appl Res Intellect Disabil. (2018) 31:e182–e200. 10.1111/jar.12291 27778426

[28] Statistics Sweden Trends and Forecasts 2017 Population, Education and Labour Market in Sweden– Outlook to Year 2035 (2017).

[29] Luthans F Avey JB Patera JL Experimental analysis of a web-based training intervention to develop positive psychological capital. Acad Manage Learn Educat. (2008) 7:209–21. 10.5465/amle.2008.32712618

[30] Maloney S Haas R Keating JL Molloy E Jolly B Sims J Effectiveness of Web-based vs. face-to-face delivery of education in prescription of falls-prevention exercise to health professionals: randomized trial. J Med Internet Res. (2011) 13:e116 10.2196/jmir.1680 22189410 PMC3278102

[31] Pusa S Dorell Å Erlingsson C Antonsson H Brännström M Sundin K. Nurses' perceptions about a web-based learning intervention concerning supportive family conversations in home health care. J Clin Nurs. (2019) 28:1314–26. 10.1111/jocn.14745 30554435 PMC7328792

[32] van der Meer L Matthews T Ogilvie E Berry A Waddington H Balandin S. Training direct-care staff to provide communication intervention to adults with intellectual disability: a systematic review. Am J Speech Lang Pathol. (2017) 26:1279–95. 10.1044/2017_AJSLP-16-0125 29084306

[33] Antonsson H Graneheim UH Isaksson U Åström S Lundström MO. Evaluation of a web-based training program for professional carers working with people with learning disabilities and challenging behavior: a pilot study with SSED-design. Issues Ment Health Nurs. (2016) 37:734–43. 10.1080/01612840.2016.1189636 27351080

[34] Hardeman W Sutton S Griffin S Johnston M White A Wareham NJ. A causal modelling approach to the development of theory-based behaviour change programmes for trial evaluation. Health Educ Res. (2005) 20:676–87. 10.1093/her/cyh022 15781446

[35] Farrell GA Shafiei T Salmon P. Facing up to ‘challenging behaviour': a model for training in staff–client interaction. J Adv Nurs. (2010) 66:1644–55. 10.1111/j.1365-2648.2010.05340.x 20497267

[36] Graneheim UH Lundman B. Qualitative content analysis in nursing research: concepts, procedures and measures to achieve trustworthiness. Nurse Educ Today. (2004) 24:105–12. 10.1016/j.nedt.2003.10.001 14769454

[37] World Medical A World medical association declaration of helsinki: ethical principles for medical research involving human subjects. JAMA. (2013) 310:2191–4. 10.1001/jama.2013.281053 24141714

[38] Lahana E Papadopoulou K Roumeliotou O Tsounis A Sarafis P Niakas D Burnout among nurses working in social welfare centers for the disabled. BMC Nurs. (2017) 16:15 10.1186/s12912-017-0209-3 28344515 PMC5364673

[39] Van Woerkom M Poell R Learning in the workplace. In: Van Woerkom M Poell R editors. Workplace Learning: Concepts, Measurement and Application. 17 Routledge (2010). p. 1–8. 10.4324/9780203850084

[40] Billett S (editor). Work, lifelong learning and cooperative education: supporting, guiding ontogenetic development. Annual Conference of New Zealand Association for Cooperative Education, Queenstown (2006).

[41] Illeris K The fundamentals of Workplace Learning: Understanding How People Learn in Working Life. Routledge (2010). 10.4324/9780203836521

[42] Brennan EM Sellmaier C Jivanjee P Grover L Is online training an effective workforce development strategy for transition service providers? Results of a comparative study. J Emotional behav Disord. (2019) 27:235–45. 10.1177/1063426618819438

[43] Lucey K The Effect of Motivation on Student Persistence in Online Higher Education: A Phenomenological Study of How Adult Learners Experience Motivation in a Web-Based Distance Learning Environment (2018).

[44] Federman J. Interruptions in online training and their effects on learning. Eur J Training Dev. (2019) 43:490–504. 10.1108/EJTD-10-2018-0100

[45] Kleinman DL Science and Technology in Society: From Biotechnology to the Internet. Malden, MA: Blackwell (2005).

[46] McCutcheon K O'Halloran P Lohan M Online learning vs. blended learning of clinical supervisee skills with pre-registration nursing students: a randomised controlled trial. Int J Nurs Stud. (2018) 82:30–9. 10.1016/j.ijnurstu.2018.02.005 29574394

[47] Wallace PE Clariana RB Achievement predictors for a computer-applications module delivered online. J Informat Syst Educat. (2020) 11:3.

[48] Fiorella L Stull AT Kuhlmann S Mayer RE Instructor presence in video lectures: the role of dynamic drawings, eye contact, and instructor visibility. J Educ Psychol. (2019) 111:1162 10.1037/edu0000325

[49] Ekman I Norberg A Swedberg K Application of person-centering in health care. In: Ekman I editor. Person-Centering in Health Care: From Philosophy to Internship. Stockholm: Liber (2014). p. 69–96.

[50] Fredriksson L Caring conversation. In: Wiklund Gustin L Bergbom I editors. Caring Science Concepts in Theory and Practice. 2 ed. Lund: Studentlitteratur AB (2017). p. 415–25.

[51] van der Meer L Nieboer AP Finkenflügel H Cramm JM. The importance of person-centred care and co-creation of care for the well-being and job satisfaction of professionals working with people with intellectual disabilities. Scand J Caring Sci. (2018) 32:76–81. 10.1111/scs.12431 28654162

[52] Damschroder LJ Aron DC Keith RE Kirsh SR Alexander JA Lowery JC. Fostering implementation of health services research findings into practice: a consolidated framework for advancing implementation science. Implementat Sci. (2009) 4:50. 10.1186/1748-5908-4-50 19664226 PMC2736161

[53] Biggs JB Tang CS-k. Teaching for quality learning at university [Elektronisk resurs] What the Student Does. Maidenhead: McGraw-Hill/Society for Research into Higher Education & Open University Press (2007).

[54] Kauffeldt A Group supervision and work- integrated learning. In: Theliander J editor. Work-Integrated Learning. Lund: Studentlitteratur (2004). p. 199–219.

[55] Harteis C Billett S Goller M Rausch A Seifried J Effects of age, gender and occupation on perceived workplace learning support. Int J Training Res. (2015) 13:64–81. 10.1080/14480220.2015.1051349

[56] Graneheim UH Lindgren B-M Lundman B. Methodological challenges in qualitative content analysis: a discussion paper. Nurse Educ Today. (2017) 56:29–34. 10.1016/j.nedt.2017.06.002 28651100

[57] Elo S Kääriäinen M Kanste O Pölkki T Utriainen K Kyngäs H Qualitative content analysis: a focus on trustworthiness. SAGE Open. (2014) 4:2158244014522633 10.1177/2158244014522633

[58] Lundman B Hällgren Graneheim U Qualitative content analysis. In: Höglund-Nielsen B Granskär M editors. Application of Qualitative Research in Health Care. 3 ed. Lund: Studentlitteratur (2017). p. 219–34.

[59] Kvale S Brinkmann S Torhell S-E The Qualitative Research Interview. Lund: Studentlitteratur (2014).

[60] Swedish Association of Local Authorities and Regions Better Health for Persons with Impairments. Swedish Association of Local Authorities and Regions (2020).

